# The population frequency of predicted pathogenic genetic variants in commonly affected CAKUT genes in the general population

**DOI:** 10.1007/s00467-026-07324-4

**Published:** 2026-05-16

**Authors:** Mary Huang, Judy Savige

**Affiliations:** The University of Melbourne Department of Medicine (Melbourne Health and Northern Health), Royal Melbourne Hospital, Parkville, VIC 3050 Australia

**Keywords:** CAKUT, Congenital anomalies of the kidney and urinary tract, Kidney development, Reflux, Kidney agenesis, Kidney atrophy, Kidney cysts

## Abstract

**Background:**

Renal imaging suggests that congenital anomalies of the kidney and urinary tract (CAKUT) affect one in 200 of the population, and 20% have a monogenic cause. This study determined the population frequency of predicted pathogenic variants in six commonly affected CAKUT genes.

**Methods:**

*HNF1B*, *SALL1*, *EYA1*, *PBX1*, *GATA3*, and *PAX2* variants were downloaded from gnomAD v.2.1.1 (*n* = 141,456) and the population frequency of predicted disease-causing variants calculated from the sum of structural, null, and predicted pathogenic missense changes in the overall cohort or from variants shared with ClinVar, HGMD, or LOVD. Population frequencies were also determined in constituent ancestries and, using our method and ClinVar assessments, in a replication cohort (gnomAD v.4.1, *n* = 807,162).

**Results:**

The population frequency of disease-causing variants in these six genes in CAKUT lies between one in 249 (our strategy) and one in 1263 (ClinVar assessments) in the gnomAD v.2.1.1 database. More than half these variants were missense changes. Predicted pathogenic variants were commonest in African-Americans (one in 149) and least common in Ashkenazim (one in 864). The population frequency estimated from gnomAD v.4.1 lies between one in 372 (our strategy) and one in 1,762 (with ClinVar).

**Conclusions:**

These calculations suggest that monogenic causes of CAKUT due to variants in these six genes are likely more common than the previously calculated one in 1,000. The ClinVar results are underestimates since assessments were not available for structural, copy number and many missense changes. However, some disease-causing variants identified here will not result in clinical disease because of incomplete penetrance and variable expressivity.

**Graphical Abstract:**

**Figure:**
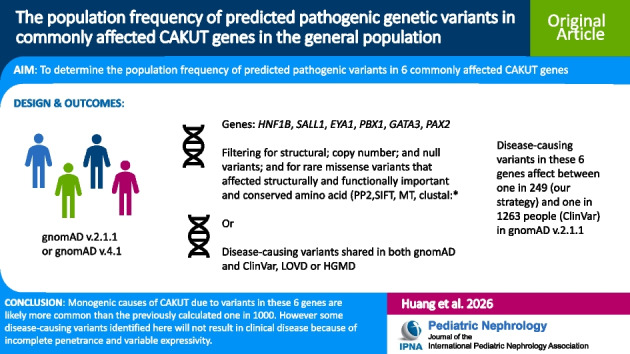
A higher resolution version of the Graphical abstract is available as Supplementary information.

**Supplementary Information:**

The online version contains supplementary material available at 10.1007/s00467-026-07324-4.

## Introduction

Congenital anomalies of the kidney and urinary tract (CAKUT) include a range of developmental malformations of the kidney and urinary tract. CAKUT is a common birth anomaly affecting 3 to 7 of every 1000 newborn [1] and is the leading cause of kidney failure in children [2, 3].

CAKUT includes ureteropelvic junction obstruction [4], kidney agenesis, dysplasia and hypoplasia, multicystic dysplastic kidneys, vesicoureteral reflux, megaureter, ectopic ureter, horseshoe kidney, and a duplex collecting system. Isolated CAKUT occurs without additional anomalies, and syndromic CAKUT has further extra-renal features [4]. Affected infants are typically diagnosed antenatally on ultrasound imaging, but those who are asymptomatic may not be identified until adolescence or later [5]. Some of those affected remain undiagnosed throughout life but may still pass on the disease-causing variant and clinical manifestations to their offspring.

CAKUT results from abnormal kidney development that is secondary to genetic, epigenetic, or environmental factors. Maternal obesity and diabetes, and possibly medications, and iron and folate deficiency increase the risk [6–8]. Monogenic causes of CAKUT have been reported in 20% of cases [9] and thus affect one in 1000 of the population, although this may be biased by being based on a cohort with typical phenotypes.

More than 150 genes are now associated with CAKUT, with many encoding transcription factors that are important in embryonic kidney development [10, 11]. Six of the commonest genes are hepatocyte nuclear factor-1B (*HNF1B*), spalt-like transcription factor 1 (*SALL1*), eyes absent homolog 1 (*EYA1*), pre-B cell leukemia (*PBX1*), GATA binding protein 3 (*GATA3*), and paired box gene 2 (*PAX2*) [12], and inheritance in all cases is autosomal dominant (https://www.omim.org/). Disease-causing variants in *HNF1B* and *PAX2* together account for at least 15% of case series of people with CAKUT [13–16]. Pathogenic variants in most of these genes have a variable phenotype and expressivity, even within families [17]. Although many more CAKUT-associated genes have been reported, most are found in only a single family [12, 18].

*HNF1B* is reported to be the commonest cause of monogenic CAKUT (MIM137920). It encodes a transcription factor of the homeodomain-containing superfamily that is essential for the development of many organs including the kidneys and urogenital tract, brain, pancreas, liver, and parathyroids [12]. Pathogenic *HNF1*B variants result in *HNF1B-*nephropathy (formerly renal cysts and diabetes syndrome, RCAD), multicystic kidney disease, focal and segmental glomerulosclerosis (FSGS), or a tubulopathy, as well as genital anomalies and monogenic diabetes (previously maturity onset diabetes of the young, MODY) [19].

*PAX2 i*s considered the second commonest cause of monogenic CAKUT [15]. *PAX2* variants are associated with renal coloboma syndrome (RCS, MIM 120330) and sometimes renal hypoplasia, multicystic dysplastic kidneys, or vesicoureteral reflux [4, 20]. Pathogenic variants are also associated with FSGS in children [21] and adults [22]. *EYA1* is a transcription factor important in development of the kidney, branchial arches, and ears [12]. Variants in *EYA1* result in the branchio-oto-renal syndrome (BOR, MIM 602588) with kidney agenesis and dysplasia in two-thirds of affected people [12], as well as ear abnormalities, hearing loss, and branchial fistulae and cysts [12]. *SALL1* loss of function variants result in Townes-Brocks syndrome (TBS, MIM 107480) [23] with renal hypodysplasia, ectopia, polycystic kidneys, and vesicoureteral reflux together with an imperforate anus, dysplastic ears and impaired hearing, and thumb abnormalities [12, 23]. *GATA3* encodes a zinc-finger transcription factor expressed in the kidneys, inner ear, and parathyroids [12]. Pathogenic *GATA3* variants are associated with hypoparathyroidism, sensorineural hearing loss, solitary kidney, renal hypodysplasia, or vesicoureteral reflux [12] (MIM 146255) [24]. *PBX1* is another transcription factor [12], and pathogenic *PBX1* variants are associated with hypoplastic and cystic kidneys, reflux, abnormal male genitalia, and sometimes congenital cardiac anomalies [1].

Previous estimates of the population frequency of CAKUT based on clinical screening have varied from one in 56 [25], 103 [26], or 627 [27, 28] to one in 2400 [29]. Having a more accurate population frequency is important to alert clinicians to the likelihood of encountering affected people, the need to examine for extrarenal manifestations, and for health service planning. Conversely, clinicians evaluating patients with extra-renal features should also be aware of the possibility of an underlying diagnosis of CAKUT.

**Fig. 1:**
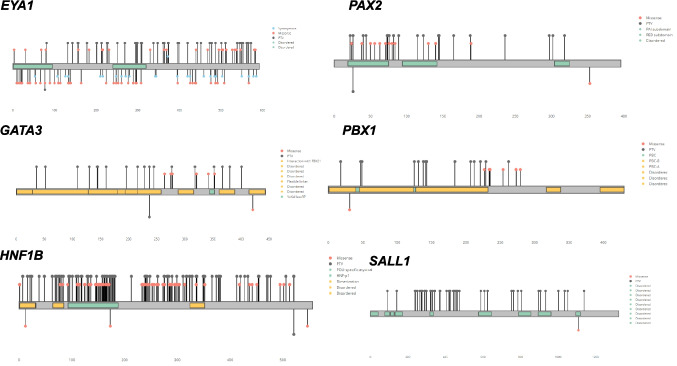
CAKUT-associated genes and distribution and type of pathogenic and likely pathogenic variants. This demonstrates that likely pathogenic and pathogenic variants according to ClinVar are distributed throughout individual genes (there are no obvious hotspots), and variants include both null and missense variants (from Simple ClinVar, https://simple-clinvar.broadinstitute.org/). This was the rationale for evaluating all null and missense variants found (except null variants in the last exon or the last 50 nucleotides of the penultimate exon)

This study calculated the population frequency of CAKUT from the number of predicted pathogenic variants in six of the commonest monogenic disease-causing genes in a cohort of normal people (Genome Aggregation Database, gnomAD v.2.1.1). Examination of pathogenic variants for these genes in Simple ClinVar (Fig. 1, https://simple-clinvar.broadinstitute.org/) suggested that variants were located throughout the genes and included both loss-of-function (null) and missense changes [12, 18]. We confirmed our results in gnomAD v.4.1 which is a much larger dataset, that includes more structural and copy number changes, and where there is only 20% overlap with gnomAD v.2.1.1. We then compared our population frequencies with those derived from gnomAD variants assessed as disease-causing in the ClinVar database (https://www.ncbi.nlm.nih.gov/clinvar), The Human Gene Mutation Database (HGMD, https://www.hgmd.cf.ac.uk/ac/index.php), or the Leiden Open Variation Database (LOVD v.3.0, https://www.lovd.nl).

**Fig. 2:**
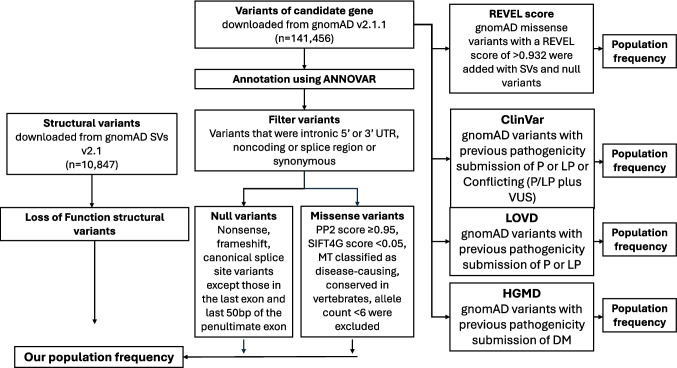
Strategy for deriving the population frequency of predicted pathogenic variants in CAKUT-associated genes in gnomAD v.2.1.1. Our population frequency was deduced from structural variants that resulted in loss of function, null variants subject to nonsense-mediated decay, and missense variants that were rare, disease-causing by all the computational tools (PP2, SIFT, MT) and affected a conserved residue. An alternative to testing for missense variants was to use REVEL. In addition, the population frequency was calculated from the number of gnomAD variants assessed as disease-causing in ClinVar, LOVD  or HGMD. Conflicting variants where the variant was reported as a VUS plus pathogenic or likely pathogenic in ClinVar were included as pathogenic. A similar strategy was used for gnomAD v.4.1 using both our strategy and ClinVar assessments.

Our approach assessed all gnomAD variants (Fig. 2) using a strategy based on the ACMG/AMP principles [30] but gnomAD has no clinical data and some benign changes may have been misinterpreted as disease-causing. Nevertheless, population frequencies using a similar, and sometimes less rigorous, strategy have been published previously for many diseases including Alport syndrome [31], Gitelman syndrome [32], autosomal dominant polycystic kidney disease [33], the mucopolysaccharidoses [34], Menke disease [35], and Fabry disease [36]. In some of these conditions, the population frequency was confirmed using an independent biochemical or histological method [31, 32].

## Methods

### gnomAD v.2.1.1 and v.4.1 variant databases

Variants in *EYA1*, *GATA3*, *HNF1B*, *PAX2, PBX1*, and *SALL1* were downloaded from gnomAD v2.1.1 (GRCh37/hg19, www.gnomAD.broadinstitute.org) and v.4.1 (GRCh38/hg38). gnomAD v.2.1.1 (*n* = 141,456) comprises whole exome sequencing (WES, *n* = 125,748) and whole genome sequencing (WGS, *n* = 15,708) data from clinical trials studying unrelated adults with diabetes,  neuropsychiatric or cardiac disease. Participants were not recruited on the basis of having a kidney disease.

gnomAD v.4.1 includes unrelated adults (*n* = 807,162), whose DNA was, in some instances, examined for structural (*n* = 63,046) or copy number (*n* = 464,297) variants too. Both gnomAD v.2.1.1 and v.4.1 included equal numbers of men and women, and their ancestries, but no clinical data. There is less than 20% overlap between the two cohorts.

gnomAD v.2.1.1 was first accessed in March 2023 and reviewed in May 2024, and gnomAD v.4.1 was reviewed in November 2024.

All participants in gnomAD had provided written informed consent for their data to be shared anonymously and available publicly for further research at the time of recruitment, so that Institutional Review Board approval was not required. 

### Annotation and filtering

Our strategy has been described previously [36]. Variants in these genes were annotated using ANNOVAR (https://annovar.openbioinformatics.org/). Variants in the intronic and 5′ or 3′ UTR or that were intronic, noncoding, splice region, or synonymous were excluded. Pathogenic and likely pathogenic intronic and other non-coding variants have been reported in CAKUT-associated genes, but were not studied here because they are rare and their accurate interpretation is difficult. Other variants were filtered according to the following approach (Fig. 2).

### Structural variants

Structural variants that were deletions and affected exons were considered pathogenic regardless of their allele counts. Duplications were not included, again because their accurate interpretation is difficult. The number of structural variants in this subset was corrected to be equivalent to the whole cohort.

### Null variants

Null variants including nonsense variants (except in the last exon and the last 50 nucleotides of the penultimate exon, which escape nonsense-mediated decay) (Supplementary Table S1), canonical splice site, and frameshift variants were classified as pathogenic regardless of their allele counts.

### Missense variants

Missense variants were “predicted pathogenic” if they were rare (allele count ≤ 5) and found to be pathogenic using all three bioinformatic tools: SIFT4G (Sorting Intolerant From Tolerant) score ≤ 0.05, PP2 (Polymorphism Phenotyping v2, PolyPhen-2) score ≥ 0.95 (http://genetics.bwh.harvard.edu/pph2/), and Mutation Taster where variants were classified as “deleterious” or (D) or (A) (MT, https://www.mutationtaster.org/info/documentation.html). Variants were also examined to determine if they affected an amino acid conserved (* or:) in vertebrates (chicken, mice, humans) using Clustal Omega (https://www.ebi.ac.uk/Tools/) and the Ensembl reference sequences (http://asia.ensembl.org/index.html).

The strategy for assessing missense variants was evaluated in two ways. The sensitivity, specificity, and positive and negative predictive values were calculated using variants that were classified as pathogenic or benign in an independent database (generally LOVD) that used clinical data in the variant evaluation (Supplementary Table S2). Our assessment for all these genes performed satisfactorily with high sensitivities (median 81%), specificities (median 100%), and positive (PPV, median 100%) and negative predictive values (NPV, median 73%) except for *SALL1* which was tested with the only four pathogenic missense variants available and identified only one of these. Secondly, REVEL (Rare Exome Variant Ensemble Learner) scores > 0.932 or > 0.80 were used to assess missense variants in the gnomAD v.2.1.1 cohort, and the number was added to the structural and null variants to obtain an independent assessment of the population frequency [37, 38]. REVEL combines the outputs of 13 individual *in silico* prediction tools (including PP2, SIFT, MT, MutPred, MutationAssessor, and phyloP among others) into a single score to improve diagnostic accuracy. A REVEL score > 0.8 has recently been demonstrated to correspond to deleteriousness [39].

### Calculation of population frequency from gnomAD

Variants downloaded from gnomAD that fulfilled all our criteria for disease-causing were classified as “Predicted pathogenic” to distinguish them from the “Pathogenic” and “Likely pathogenic” terms used by the ACMG/AMP criteria [30]. Population frequencies of the six autosomal dominant CAKUT genes were calculated using the average allele count and total number of people examined for each gene. The population frequencies for these genes were estimated assuming that each person included in gnomAD had only one genetic variant for CAKUT, while also recognizing that sometimes variants are found in more than one gene [40].

### Predicted pathogenic variants for CAKUT in people of different ancestries

Population frequencies of predicted pathogenic variants were then calculated for people of each ancestry included in this version of gnomAD.

### Population frequencies of CAKUT using different databases

All gnomAD variants were also examined in ClinVar, HGMD, and LOVD databases for previous reports of pathogenicity. The population frequencies for gnomAD variants that were pathogenic, likely pathogenic, or conflicting (where different assessments included a VUS and pathogenic or likely pathogenic) were then calculated for ClinVar, and the population frequencies for pathogenic variants in HGMD and LOVD were also calculated. Disease-causing variants in these databases were included regardless of the number of times they were found in gnomAD.

### Population frequency of CAKUT of gnomAD v.4.1 using our strategy and ClinVar

We then repeated the population frequency calculations using our approach and gnomAD v.4.1 including structural (*n* = 63,046) and copy number variants (*n* = 464,297) both corrected (× 13, × 1.7 respectively) for the whole cohort, as well as null and missense changes. In addition, we assessed the population frequency of CAKUT from gnomAD v.4.1 using variants assessed as pathogenic or likely pathogenic or conflicting (P/LP/VUS) in ClinVar as described above.

### Statistical analysis

Results were compared using chi-squared testing (https://www.graphpad.com/quickcalcs/).

## Results

### Population frequency of CAKUT from our strategy of gnomAD v.2.1.1

Overall, there were 273 variants in one of the six CAKUT genes in 461 people from gnomAD v.2.1.1 that predicted pathogenic. This was equivalent to a population frequency for CAKUT of 461 in 114,963 or one in 249 people (Table 1).

**Table 1:** Population frequencies of predicted pathogenic variants in CAKUT-associated genes in gnomAD v.2.1.1

	*HNF1B* (*n* = 119,441 people)	*SALL1* (*n* = 120,389 people)	*EYA1* (*n* = 120,908 people)	*PBX1* (*n* = 98,815 people)	*GATA3* (*n* = 109,227 people)	*PAX2* (*n* = 120,946 people)	Total (*n* = 114,963 people)
Structural variants (*n* = 10,847)	One variant in 3 people, 33 people corrected for whole cohort	None	None	None	None	None	One variant in 33 people
Null variants	2 variants in 2 people	One variant in 1 person	8 variants in 8 people	3 variants in 3 people	3 variants in 3 people	8 variants in 23 people	25 variants in 40 people
Missense variants	27 variants in 39 people	114 variants in 186 people	50 variants in 84 people	8 variants in 9 people	17 variants in 27 people	31 in 43 people	247 variants in 388 people
Total	30 variants in 74 people	115 variants in 187 people	58 variants in 92 people	11 variants in 12 people	20 variants in 30 people	39 variants in 66 people	273 variants in 461 people
Population frequency	One variant in 1,614 people	One variant in 644 people	One variant in 1,314 people	One variant in 8,234 people	One variant in 3,641 people	One variant in 1,832 people	One variant in 249 people

^1^Structural variants corrected for smaller whole genome sequencing cohort × 13. This analysis does not include copy number variants which were not available for gnomAD v.2.1.1

^2^Null variants including < 6 alleles but not in the last exon or last 50 nucleotides of the second last exon.

^3^Total pathogenic variants positive in all, < 6 alleles

Variants in the *SALL1* gene were the commonest predicted pathogenic variants (one in 644) followed by changes in *EYA1* (one in 1,314) or *HNF1B* (one in 1,614). Deletions occurred in nearly half the people with *HNF1B* variants [19], and the number of *HNF1B* structural changes after correction (*n* = 33) was nearly equivalent to the combined number of missense and null variants  (*n* = 41). No pathogenic structural variants were found in the other five genes. In general, more people were found to have missense (*n* = 388) than other changes (*n* = 73) using our strategy.

When a REVEL score > 0.932 was used to assess the missense variants in gnomAD v.2.1.1, structural, null, and predicted pathogenic missense variants were found in 188 people corresponding to a population frequency of one in 611 (188/114,963) (*p* < 0.0001 compared with our assessment) (Table 2). REVEL levels > 0.932 may have been too stringent and resulted in an underestimate for the population frequency. When a REVEL score > 0.8 [39] was used instead, predicted pathogenic variants were found in 319 people corresponding to a population frequency of one in 360 (319/114,963).

**Table 2:** Population frequency of predicted pathogenic variants in CAKUT-associated genes in gnomAD v.2.1.1 using various databases

	*HNF1B* (*n* = 119,441)	*SALL1* (*n* = 120,389)	*EYA1* (*n* = 120,908)	*PBX1* (*n* = 98,816)	*GATA3* (*n* = 109,277)	*PAX2* (*n* = 120,946)	Total (*n* = 114,963)	Population frequency (*n* = 114,963)
Our assessment	30 variants in 74 people	115 variants in 187 people	58 variants in 92 people	11 variants in 12 people	20 variants in 30 people	39 variants in 66 people	273 variants in 461 people	461/114,963 or one in 249
REVEL > 0.932 (missense only)	5 variants in 9 people	2 variants in 2 people	6 variants in 12 people	None	4 variants in 5 people	8 variants in 17 people	25 variants in 45 people	45/114,963 or one in 2,555
REVEL (SV and CNV, null and missense variants)	36 variants in 114 people	3 variants in 3 people	14 variants in 20 people	3 variants in 3 people	7 variants in 8 people	16 variants in 40 people	78 variants in 155 people	188/114,963 or one in 612
ClinVar	13 variants in 61 people	3 variants in 5 people	5 variants in 6 people	1 variant in one person	2 variants in 5 people	2 variants in 13 people	26 variants in 91 people	91/114,963 or one in 1,263
HGMD	27 variants in 1,179 people	11 variants in 1,156 people	12 variants in 430 people	1 variant in one person	1 variant in one person	6 variants in 85 people	58 variants in 2,852 people	2852/114,963 or one in 40
LOVD	6 variants in 12 people	None	9 variants in 119 people	None	1 variant in one person	2 variants in 131 people	18 variants in 263 people	263/114,963 or one in 437

*N* = total number in cohort examined for variants in this gene

*SV* structural variants, *CNV* copy number variants

**Fig. 3:**
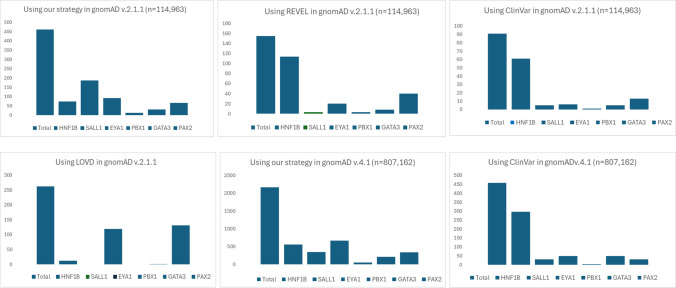
Numbers of predicted pathogenic variants identified in the six CAKUT genes in gnomAD v.2.1.1 and v.4.1 using different strategies. These bar graphs demonstrate the numbers of predicted pathogenic variants for each of the six genes examined

When gnomAD variants were examined for those found pathogenic or likely pathogenic in ClinVar, there were variants in 91 people corresponding to a population frequency of one in 1,263 (*p* < 0.0001 compared with our assessment) (Table 2, Fig. 3). While ClinVar assessments are considered accurate, they were not available for all gnomAD variants so that the calculated population frequency was an underestimate. In particular, there were no ClinVar assessments for structural variants. If the number of structural variants found disease-causing in our assessment were added to the ClinVar assessments, then there would be 124 variants in 114,963 people or one in 923.

However, the number of assessments in ClinVar for these genes in gnomAD v.2.1.1 was limited, which suggested that the strategy using ClinVar underestimated the population frequency of disease-causing variants. ClinVar assessments were available for 78/273 variants in *HNF1B* (29%), 131/786 in *SALL1* (17%), 76/310 in *EYA1* (25%), 6/126 in *PBX1* (5%), 33/214 in *GATA3*, and 56/229 in *PAX2* (24%), that is, with a median of 21%, range 5–29% for gnomAD v.2.1.1.

When gnomAD v.2.1.1 variants were examined for those also found in HGMD, there were 2,852 variants corresponding to a population frequency of one in 40 (Table 2, Fig. 3). When pathogenic variants in LOVD were used to examine gnomAD v.2.1.1, there were 263 variants corresponding to a population frequency of one in 437 (Table 2, Fig. 3).

**Fig. 4:**
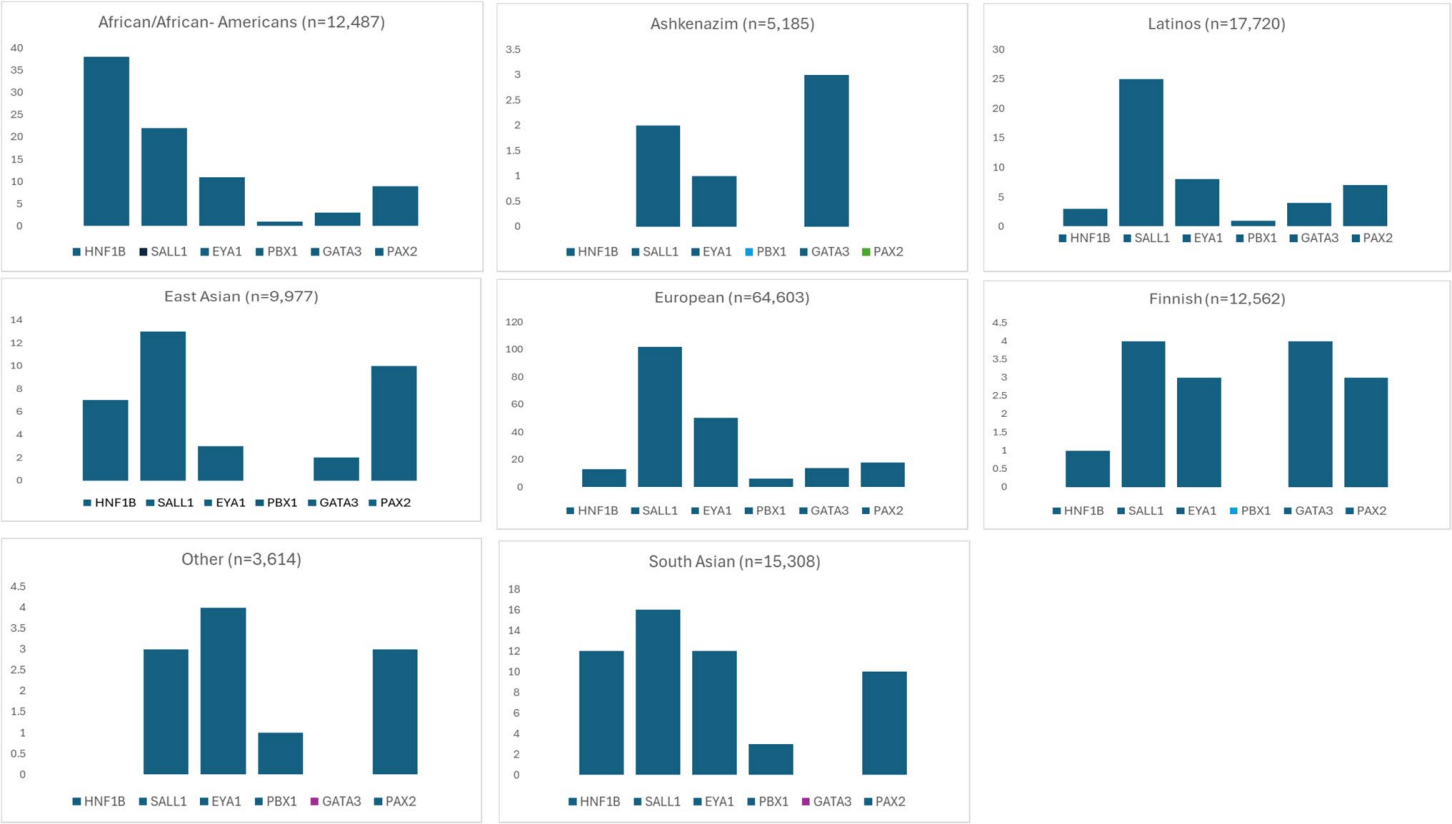
Numbers of predicted pathogenic variants identified in the six CAKUT genes in gnomAD v.2.1.1 in people of different ancestries. These bar graphs demonstrate the numbers of predicted pathogenic variants for each of the six genes examined  in people of different ancestries (African/African-American, Latinos, Ashkenazim, East Asian, European, Finnish, South Asian, and Other). Structural variants were only identified in people of African/African-American ancestry

When our predicted pathogenic variants were examined in people of different ancestries, they were commonest in people of African/American ancestry (84/12,487, or one in 149, mainly due to structural changes) and East Asians (41/9,977, one in 243) and least common in Finnish people (15/12,562, one in 837 people) and Ashkenazim (6/5,185 or one in 864) (Table 3, Fig. 4). These calculations included structural, null, and missense changes but not copy number variants.

**Table 3:** Predicted pathogenic variants in the CAKUT-associated genes in people of differing ancestries

		*HNF1B* (*n* = 119,441)	*SALL1*** (***n*** =** 120,389)	*EYA1* (*n* = 120,908)	*PBX1* (*N* = 98,815)	*GATA3* (*n* = 109,227)	*PAX2* (*n* = 120,946)	Total (*n* = 114,963)	Population frequency in this ancestry
**African American (*n* = 12,487)**	SV	33	0	0	0	0	0	33	84/12,487 or one in 149
	Null	0	0	1	1	0	0	2	
	Missense	5	22	10	0	3	9	49	
**Latino (*n* = 17,720)**	Null	0	0	2	1	0	4	7	48/17,720 or one in 369
	Missense	3	25	6	0	4	3	41	
**Ashkenazim (*n* = 5,185)**	Null	0	0	0	0	1	0	1	6/5,185 or one in 864
	Missense	0	2	1	0	2	0	5	
**East Asian (*n* = 9,977)**	Null	1	0	0	0	0	10	1	41/9,977 or one in 243
	Missense	6	13	3	0	2	6	30	
**European (*n* = 64,603)**	Null	1	0	5	0	1	5	12	203/64,603 or one in 318
	Missense	12	102	45	6	13	13	191	
**Finnish (*n* = 12,562)**	Null	0	1	0	0	1	0	2	15/12,562 or one in 837
	Missense	1	3	3	0	3	3	13	
**South Asian (*n* = 15,308)**	Null	0	0	0	0	0	2	2	53/15,308 or one in 289
	Missense	12	16	12	3	0	8	51	
**Other (*n* = 3614)**	Null	0	0	0	0	0	2	2	11/3,614 or one in 329
	Missense	0	3	4	1	0	1	9	

This analysis does not include copy number variants which were not available for gnomAD v.2.1.1. Data are available for Finnish people separate from non-Finnish Europeans in gnomAD

SV structural variants

### Population frequency of CAKUT from our approach and ClinVar assessment of gnomAD v.4.1

gnomAD v4.1 variants were evaluated using our assessment and the ClinVar assessment based on previous reported results. gnomAD v.4.1 had the advantage of including structural and copy number variants in some patients. Our assessment found 733 predicted pathogenic variants in 2,168 people, corresponding to a population frequency of one in 372 people (*p* < 0.0001 compared with our estimate in gnomAD v.2.1.1) (Table 4).

**Table 4:** Predicted pathogenic variants in gnomAD v.4.1 in the 6 CAKUT-associated genes using our strategy and ClinVar assessments

	*HNF1B*	*SALL1*	*EYA1*	*PBX1*	*GATA3*	*PAX2*	Total
**Our strategy**							
**Structural variants (*n* = 63,046)** (× 13, corrected for whole cohort)	One LoF variant in 7 people (91 people)	No variants	3 LoF variants in 12 people (156 people)	No variants	One LoF variant in 6 people (78 people)	2 LoF variants in 6 people (78 people)	7 LoF variants in 403 people
**Copy number variants (*n* = 464,297)** (× 1.7, corrected for whole cohort)	7 variants in 154 people (268 people)	No variants	6 variants in 6 people (10 people)	2 variants in 2 people (3 people)	3 variants in 5 people (9 people)	1 variant in 2 people (3 people)	19 variants in 293 people
**Null variants**	14 variants in 31 people	21 variants in 28 people	55 variants in 313 people	11 variants in 12 people	17 variants in 32 people	49 variants in 123 people	167 variants in 539 people
**Missense variants**	83 variants in 165 people	212 variants in 323 people	101 variants in 190 people	21 variants in 34 people	44 variants in 90 people	89 variants in 131 people	550 variants in 933 people
**Our assessment (*n* = 807,162)**	105 variants in 555 people	233 variants in 351 people	156 variants in 669 people	34 variants in 49 people	64 variants in 209 people	141 variants in 335 people	733 variants in 2,168 people
**Population frequency of our assessment**	555/807,162 or one in 1454	351/807,162 or one in 2,300	669/807,162 or one in 1,207	49/807,162 or one in 16,473	209/807,162 or one in 3,862	335/807,162 or one in 2,409	2168/807,162 or one in 372
**ClinVar assessment**							
**Pathogenic or Likely pathogenic variants in ClinVar (*n* = 807,162)**	30 variants in 296 people	7 variants in 31 people	12 variants in 49 people	3 variants in 3 people	8 variants in 49 people	11 variants in 30 people	71 variants in 458 people
**Population frequency of pathogenic or likely pathogenic variants in ClinVar**	296/807,162 or one in 2,726	31/807,162 or one in 26,037	49/807,162 or one in 16,472	3/807,162 or one in 269,054	49/807,162 or one in 16,473	30/807,162 or one in 26,905	458/807,162 or one in 1,762

With our assessment, the commonest affected genes were *EYA1* (one in 1,207), *HNF1B* (one in 1,454), and *SALL1* (one in 2,300). The frequencies of *EYA1* (chi squared = 0.193, *p* = 0.66) and *HNF1B* variants (chi squared = 0.257, *p* = 0.61) were not different from those found in gnomAD v.2.1.1, but disease-causing *SALL1* variants were much less common in gnomAD 4.1 (one in 2,300, chi-squared = 63.94, *p* < 0.0001).

With the ClinVar assessment, there were 71 pathogenic or likely pathogenic or conflicting (P/LP/VUS) variants in 458 people corresponding to a population frequency of one in 1,762 (*p* < 0.0001 compared with our estimate in gnomAD v.2.1.1). Again, there were no ClinVar assessments for structural or copy number changes. If all the structural and copy number variants included in our assessment were added to the total ClinVar variants assessed as disease-causing then the total number of variants (1,154 in 807,162 people) is equivalent to a population frequency of one in 699.

However, ClinVar assessments were only available in gnomAD v.4.1 for 162/679 variants in *HNF1B* (24%), 259/1,774 in *SALL1* (15%), 124/778 in *EYA1* (16%), 3/348 in *PBX1* (1%), 15/577 in *GATA3* (3%), and 17/678 in *PAX2* (3%). Thus, while gnomAD v.4.1 included more structural and copy number variants than gnomAD v.2.1.1, the median number of ClinVar assessments for its variants was 9%, range 1–24% compared with a median of 21%, and range 5–29% for gnomAD v.2.1.1. This means that, again, the population frequencies deduced from ClinVar assessments were underestimates and that the population frequency deduced from gnomAD v.4.1 was likely to represent a greater underestimate than for gnomAD v.2.1.

## Discussion

These studies suggest that the population frequency of predicted pathogenic variants in six of the commonest CAKUT genes lies in the range between one in 249 and one in 1263. However, the number of people with CAKUT-associated clinical features will be less than this because of reduced penetrance and variable expressivity.

According to our strategy, the commonest affected CAKUT genes in gnomAD v.2.1.1 were *SALL1*, *EYA1*, and *HNF1B,* whereas in gnomAD v.4.1, they were *EYA1*, *HNF1B*, and *SALL1.* This is different from published series that have found *HNF1B* and *SALL1* most often and may be due in part to the large number of structural and copy number variants detected in *HNF1B* and *EYA1* in gnomAD v.4.1 [13–16]. However, if confirmed, *SALL1* variants may be less penetrant and associated with fewer CAKUT features, meaning that they are often overlooked. Overall, missense variants were more common than null changes in these six genes and, in general, missense variants may be associated with less penetrant disease [41]. However, there are also exceptions such as the 17q12 deletion that has a better prognosis for kidney disease than *HNF1B* missense changes [42].

Our assessment suggested that predicted pathogenic variants in these six CAKUT genes were more common in people of African/African-American ancestry, in part from the correction for the smaller cohort of the three *HNF1B* structural variants in gnomAD v.2.1.1. This result confirms global reports of CAKUT being more common in African countries although socioeconomic factors may also contribute [43]. CAKUT variants were also common in people of an East Asian background. Predicted pathogenic variants were least common in Ashkenazim and Finns, which may result from their relative social and geographic isolation.

However, there were a number of methodological considerations in assessing the results from these studies. Only six CAKUT genes were examined, whereas more than 150 have been identified. gnomAD v.2.1.1 underrepresents people with severe or early onset disease, such as those with CAKUT-associated kidney failure who were ineligible for the clinical trials that represented most of this cohort. The majority of gnomAD v.2.1.1 samples were tested by WES which detected structural changes in only a subset and did not examine for copy number changes. This was particularly important for *HNF1B* where half the variants are large deletions [19]. Interestingly, no structural changes were found in the other five genes in gnomAD v.2.1.1.

While our analytical strategy had the advantage that it provided an assessment for each variant and our criteria for pathogenicity were more stringent than those used previously in similar studies [32, 33], its major source of inaccuracy was in evaluating missense changes. This was demonstrated by the low sensitivity for *SALL1* variants (Suppl Table S2) and by comparison with the highly rigorous REVEL assessment.

ClinVar assessments are generally accurate [44] because they are submitted from accredited testing laboratories who use sequencing from people with clinically suspected kidney disease, as well as the ACMG/AMP criteria and variant rarity based on recently available large datasets. However, ClinVar also overrepresents high-penetrance variants from clinically referred cohorts. In addition, ClinVar only included assessments for 21% of gnomAD v.2.1.1 variants in these six genes (median, range 5–29%) and not for structural or copy number changes. There were even fewer assessments for gnomAD v.4.1 (median variants assessed 9%, range 1–24%). The low number of ClinVar assessments is explained by fewer laboratories performing genetic testing for CAKUT because of its mainly clinical diagnosis, the large number of affected genes, the difficulty in assessing missense changes, and the lack of treatment. This suggests that the population frequency of the monogenic forms of CAKUT is more common than one in 1,263 and possibly closer to our estimate of one in 249. The population frequencies derived from the LOVD and HGMD databases have the advantage of largely being reported from people with the clinical features of CAKUT, but unlike ClinVar, they comprise many variants reported before the availability of ACMG/AMP guidelines and large datasets for checking variant rarity. LOVD recorded no variants at all for *SALL1* and *PBX1.*

Large biobanks that include both genomic and clinical data are not necessarily helpful in obtaining more accurate population frequencies. They are still limited by our inability to accurately assess variants for pathogenicity, and CAKUT will not be suspected in many people recruited, who will not have undergone imaging to confirm kidney disease. Others may have pathogenic variants but incompletely penetrant disease without an obvious CAKUT phenotype. Some diseases such as reflux nephropathy may have resolved in adulthood. Indeed, LOVD and HGMD both include clinical information, but their inaccuracy lies in determining whether the genetic variant is responsible for the phenotype.

The strengths of this study were the large cohorts who had undergone genetic testing, the rigorous criteria used for our variant assessment, the comparison of multiple strategies, the use of gnomAD v.4.1 as a replication cohort, and the ability to determine population frequencies in people of different ancestries.

However this study also had a number of limitations. The gnomADv.2.1.1 population is not normal. Just more than half of the study participants have cardiac or neuropsychiatric disease, or diabetes, and the others are age- and sex-matched controls. gnomAD v.4.1 does not exclude participation based on phenotype and thus is expected to include people with rare diseases. None of the participants was chosen on the basis of having or not having kidney disease. Further limitations were the inability to confirm variant pathogenicity because of the lack of clinical data in gnomAD, the lack of ClinVar assessments for all gnomAD variants, the incompleteness of the structural and copy number analysis, and the potential inaccuracies in our computational assessments. In addition, we only examined six of the many CAKUT-associated genes; sometimes, pathogenic variants in two CAKUT genes are found in the same individual, and we did not consider the possibility of disease due to mosaicism. Nevertheless, we have used this approach previously to estimate the population frequencies of Alport syndrome and monogenic diseases, such as Fabry disease and autosomal dominant polycystic kidney disease [31, 45], and all the calculated population frequencies for CAKUT suggested that these diseases were more common than previously believed.

In conclusion, this study demonstrated that predicted pathogenic variants found in gnomAD in six CAKUT genes affect between one in 249 and one in 1,263 people. Our assessment of one in 249 as the population frequency for CAKUT disease-causing variants in gnomAD is likely to be an overestimate because of the difficulty in assessing missense changes. In contrast, the observation of one in 1,263 deduced from ClinVar data is likely to be an underestimate because ClinVar assessments were not available for all gnomAD changes. Previous studies have found that CAKUT affects one in 200 people of whom one in 5, or overall one in 1,000, have a monogenic cause. Our population frequency is consistent with a greater proportion of people with CAKUT having a genetic basis. Importantly, about half the predicted pathogenic variants in the CAKUT genes were missense changes that are commonly, but not always, associated with a milder phenotype [41], so that the number of people with clinical features of CAKUT is likely to be less common than our estimate of one in 249.

Future improvements in computational tools and the use of large databanks with both genomic and clinical data will enable more accurate estimates of the population frequency of CAKUT from these six genes as well as disease penetrance.

## Supplementary Information

Below is the link to the electronic supplementary material.

ESM 1 Graphical abtsract (PPTX 84.8 KB)

ESM 2 (PDF 101 KB)

## Data Availability

All the data reported here is either available in the manuscript or available from the authors on reasonable request.
