# Supplementary material for: The population frequency of predicted pathogenic genetic variants in commonly affected CAKUT genes in the general population
Source: Pediatr Nephrol. 2026 May 16;41(10):3519–30. doi: 10.1007/s00467-026-07324-4 (PMC13481559; doi:10.1007/s00467-026-07324-4)
Supplement: Supplementary file 1 [file 467_2026_7324_MOESM2_ESM.pdf]

Supplementary Tables

Supplementary Table S1: Location of cut-off for nonsense mediated decay from Alamut
Supplementary Table S2: Accuracy of our strategy for missense variants in the CAKUT-associated genes

Table S1: Location of cut-off for nonsense mediated decay from Alamut

	MANE select transcript	Position of second last exon	Last 50bp of the penultimate exon
HNF1B	NM_000458.4	c.1535 → c.1653	c.1603
SALL1	NM_002968.3	c.77 → c.3534	c.3484
EYA1	NM_000503.6	c.1598 → c.1698	c.1648
PBX1	NM_002585.4	c.1111 → c.1200	c.1150
GATA3	NM_001002295.2	c.925 → c.1050	c.1000
PAX2	NM_000278.5	c.1022 → c.1108	c.1058

The cut off for nonsense-mediated decay was calculated from the location of the nucleotide 50 nt in from the end of the penultimate exon according to the MANE reference sequence

Table S2: Accuracy of our strategy for missense variants in the CAKUT-associated genes

	Benign	Pathogenic	Sensitivity	Specificity	PPV	NPV
HNF1B	ClinVar n=4<br>gnomAD[1] n=14	ClinVar n=20<br>HGMD n=15<br>LOVD n=13	45/48 (94%)	18/18 (100%)	45/45 (100%)	18/21 (86%)
SALL1	ClinVar n=20<br>LOVD n=2<br>gnomAD[1] n=22	ClinVar n=2<br>HGMD n=1<br>LOVD n=1	1/4 (25%)	38/44 (86%)	1/7 (14%)	38/41 (93%)
EYA1	ClinVar n=8<br>LOVD n=1<br>gnomAD[1] n=9	ClinVar n=15<br>HGMD n=3<br>LOVD n=15	25/33 (76%)	17/18 (94%)	25/26 (96%)	17/25 (68%)
PBX1	ClinVar n=2<br>gnomAD[1] n=2	ClinVar n=13<br>HGMD n=4<br>LOVD n=1	13/18 (72%)	4/4 (100%)	13/13 (100%)	4/9 (44%)
GATA3	ClinVar n=2<br>LOVD n=0<br>gnomAD[1] n=3	ClinVar n=15<br>HGMD n=11<br>LOVD n=17	37/43 (86%)	5/5 (100%)	37/37 (100%)	5/11 (45%)
PAX2	ClinVar n=1<br>LOVD n=3<br>gnomAD[1] n=6	ClinVar n=19<br>HGMD n=12<br>LOVD n=19	47/50 (94%)	10/10 (100%)	47/47 (100%)	10/13 (77%)

[1]Variants from gnomAD with allele count >30 presumed benign
NPV – negative predictive value, PPV – positive predictive value
